# Prevalence and correlates of e-cigarette use among older adults aged 60 years and over

**DOI:** 10.1007/s41999-026-01539-2

**Published:** 2026-06-29

**Authors:** Yusuff Adebayo Adebisi, Anas Ali Alhur, Isaac Olushola Ogunkola, Najim Z. Alshahrani, Don Eliseo Lucero-Prisno, Usman A. Adeniran, Giulio Geraci, Venera Tomaselli, Riccardo Polosa

**Affiliations:** 1 College of Social Sciences, University of Glasgow, Glasgow, UK; 2 Department of Clinical & Experimental Medicine, University of Catania, Catania, Italy; 3 Department of Health Informatics, College of Public Health and Health Informatics, University of Hail, Hail, Saudi Arabia; 4 Nuffield Department of Population Health, University of Oxford, Oxford, UK; 5 Department of Family and Community Medicine, Faculty of Medicine, University of Jeddah, Jeddah, Saudi Arabia; 6 Department of Global Health and Development, London School of Hygiene and Tropical Medicine, London, UK; 7 King Fahd University, Dhahran, Saudi Arabia; 8 Faculty of Medicine and Surgery, “Kore” University of Enna, Enna, Italy; 9 Center of Excellence for the Acceleration of HArm Reduction (CoEHAR), University of Catania, Catania, Italy; 10 Centre for the Prevention and Treatment of Tobacco Addiction (CPCT), University Teaching Hospital “Policlinico-S.Marco”, University of Catania, Catania, Italy

**Keywords:** E-cigarettes, Vaping, Older adults, Geriatric, Prevalence, Smoking, Harm reduction, Scotland, Health survey

## Abstract

**Purpose:**

E-cigarette use has increased substantially in the general population, yet evidence on use among older adults remains limited. Older adults bear a disproportionate burden of smoking-related morbidity and, in some populations, have shown persistently high smoking prevalence relative to younger age groups. Understanding the prevalence and correlates of e-cigarette use in this population is essential to inform targeted tobacco control and harm reduction strategies.

**Methods:**

We conducted a pooled cross-sectional analysis of 13,297 adults aged 60 years and over (mean age 71.4 years; 54.3% female) from seven waves (2017–2024, excluding 2020) of the Scottish Health Survey (SHeS), a nationally representative household survey. Current e-cigarette use was the primary outcome. Survey-weighted logistic regression was used to estimate crude and adjusted odds ratios for the association between current e-cigarette use and sociodemographic, behavioural, and health-related covariates, accounting for the complex survey design.

**Results:**

The weighted prevalence of current e-cigarette use among older adults was 4.5% (95% CI: 4.1–4.9). Prevalence remained broadly stable across survey years, ranging from 2.16% (95% CI: 1.43–3.25) in 2021 to 5.55% (95% CI: 4.43–6.93) in 2023. E-cigarette use was overwhelmingly concentrated among those with a smoking history: current smokers (adjusted OR 18.78, 95% CI: 11.79–29.92, *p* < 0.001) and ex-smokers (adjusted OR 20.17, 95% CI: 13.10–31.06, *p* < 0.001) had markedly elevated odds compared with never smokers. E-cigarette use declined sharply with advancing age: compared with the 60–64 reference group, adjusted odds were lower at ages 65–69 (OR 0.61, 95% CI: 0.47–0.79, *p* < 0.001), 70–74 (OR 0.44, 95% CI: 0.33–0.58, *p* < 0.001), 75–79 (OR 0.19, 95% CI: 0.13–0.29, *p* < 0.001), and 80 and over (OR 0.11, 95% CI: 0.06–0.19, *p* < 0.001). Residence in less deprived areas was associated with lower odds of use compared with the most deprived quintile: fourth quintile (OR 0.49, 95% CI: 0.34–0.70, *p* < 0.001) and least deprived (OR 0.41, 95% CI: 0.27–0.61, *p* < 0.001). Respondents reporting fair (OR 1.32, 95% CI: 1.03–1.70, *p* = 0.028) or bad/very bad self-rated health (OR 1.73, 95% CI: 1.31–2.27, *p* < 0.001) had higher odds than those in very good or good health. Being divorced was independently associated with current use (OR 1.65, 95% CI: 1.22–2.23, *p* = 0.001). Sex and alcohol consumption were not significant correlates.

**Conclusions:**

E-cigarette use among Scottish older adults was low but non-trivial and was strongly patterned by smoking history. The concentration of use among current and former smokers suggests that e-cigarette use in this age group occurs mainly among people with prior exposure to combustible tobacco, although the cross-sectional design cannot determine whether use reflects cessation, substitution, experimentation, or persistent dual use. The steep age gradient, deprivation patterning, and association with poorer self-rated health highlight the need for age-sensitive clinical counselling and balanced risk communication in older adults.

**Supplementary Information:**

The online version contains supplementary material available at https://doi.org/10.1007/s41999-026-01539-2.

## Key summary points

**Aim:**

To estimate the prevalence and identify the independent correlates of current e-cigarette use among adults aged 60 years and over in Scotland using nationally representative data from the Scottish Health Survey (2017–2024).

**Findings:**

The weighted prevalence of current e-cigarette use was 4.5%, remained broadly stable over the study period, and was overwhelmingly concentrated among current and former cigarette smokers. E-cigarette use declined sharply with advancing age, was higher in more deprived areas, and was associated with poorer self-rated health, while sex and alcohol consumption were not significant independent correlates.

**Message:**

E-cigarette use among Scottish older adults was low but concentrated among people with a history of cigarette smoking. This pattern is compatible with use after smoking initiation rather than primary nicotine uptake; however, the cross-sectional design cannot determine motivation, timing, or whether e-cigarettes were used for cessation, substitution, or persistent dual use. Findings highlight the need for age-sensitive clinical counselling, balanced risk communication, and routine assessment of nicotine product use in older adults, particularly those with multimorbidity, frailty, or poor self-rated health.

**Supplementary Information:**

The online version contains supplementary material available at https://doi.org/10.1007/s41999-026-01539-2.

## Introduction

Tobacco smoking remains a leading preventable cause of morbidity and mortality worldwide, accounting for an estimated eight million deaths annually [1]. Among older adults, smoking confers a particularly heavy burden: it accelerates age-related physiological decline, exacerbates chronic disease, and compounds the vulnerability associated with frailty and multimorbidity [2, 3]. In Scotland, tobacco control policy has achieved substantial reductions in overall smoking prevalence over the past two decades, yet progress has not been uniform across age groups [4].

The persistence of smoking among older adults reflects a convergence of factors. Longer smoking histories produce deeper nicotine dependence, and repeated unsuccessful quit attempts may engender feelings of futility [5, 6]. Older adults are less likely to be offered smoking cessation support by healthcare providers, and quit rates among those who do attempt cessation are lower than in younger populations [7–10]. The clinical consequences of continued smoking in later life are substantial: among older adults who smoke, approximately one in four report a smoking-related cancer and nearly half report another smoking-related chronic disease [11, 12]. These figures underline the urgency of identifying effective strategies to reduce the harm of continued tobacco use in ageing populations.

E-cigarettes have emerged as a potentially disruptive technology in the landscape of tobacco harm reduction [13]. By delivering nicotine through an aerosolised liquid rather than combusted tobacco, e-cigarettes expose users to substantially fewer toxicants than conventional cigarettes [14]. Randomised controlled trials and population-level studies suggest that e-cigarettes are more effective than nicotine replacement therapy for smoking cessation [15, 16], and public health agencies in the United Kingdom have adopted a broadly favourable stance towards their use as a quit aid [17]. However, the adoption of e-cigarettes has not been uniform across the age spectrum. Despite the clear public health significance of this question, the evidence base on e-cigarette use among older adults remains underdeveloped [18]. Most population-level studies of e-cigarette use have focused on younger adults or the general adult population, and those that have examined age differences have typically treated older adults as a residual category rather than as the primary analytic focus. Dedicated research on the prevalence, patterns, and correlates of e-cigarette use in older populations is needed to inform age-appropriate tobacco control strategies, guide clinical counselling, and address the widening age-related disparity in smoking cessation outcomes.

This study addresses this gap using data from the Scottish Health Survey (SHeS), a nationally representative household survey that provides detailed information on tobacco and nicotine product use, sociodemographic characteristics, and health status. The aims of this study are twofold: first, to estimate the prevalence of current e-cigarette use among adults aged 60 years and over in Scotland, including trends over time and variation by sociodemographic and health characteristics; and second, to identify the independent correlates of current e-cigarette use in this population using multivariable logistic regression, with a particular focus on the role of cigarette smoking history.

## Methods

### Study design, data source, and study population

This study employed a pooled cross-sectional design using individual-level data from the 2017, 2018, 2019, 2021, 2022, 2023, and 2024 waves of the Scottish Health Survey (SHeS). The SHeS is a nationally representative household survey commissioned by the Scottish Government to monitor the health and health-related behaviours of people living in Scotland [19]. It provides population-level evidence to support public health surveillance, policy development, and the evaluation of health interventions. The survey uses a multistage, stratified probability sampling design to ensure broad representativeness across age, sex, socioeconomic circumstances, and geographic areas. Each annual wave is independently sampled, making the SHeS a repeated cross-sectional survey rather than a longitudinal cohort. The 2020 wave was excluded because the COVID-19 pandemic disrupted routine data collection procedures, limiting comparability with the other survey years [20].

A total of 44,996 respondents participated across the seven survey years included in this analysis: 5,300 in 2017; 6,790 in 2018; 6,881 in 2019; 6,157 in 2021; 6,158 in 2022; 7,133 in 2023; and 6,577 in 2024. Data were collected using structured computer-assisted interviews, with self-completion options for more sensitive questions where appropriate. For the present analysis, pooled harmonised individual-level datasets were used to ensure consistency in coding and measurement across survey years.

The analytic sample was restricted to adults aged 60 years and over, consistent with the United Nations definition of older adults and the study focus on the geriatric population. This age threshold is endorsed by both the World Health Organization and the United Nations and has been adopted in international guidance on the protection and health needs of older persons [21, 22]. The restriction reduced the pooled sample from 44,996 to 13,316 age-eligible respondents after excluding those younger than 60 years and those with invalid or missing age values. Respondents with non-substantive responses to the e-cigarette use item, including “not applicable” and “schedule not applicable”, were excluded, yielding a final analytic sample of 13,297 older adults.

### Outcome

The outcome was current e-cigarette use. Respondents were classified using the harmonised SHeS variable on use of e-cigarettes or vaping devices, which distinguishes current use, past use, and never use. For the primary analysis, this variable was recoded into a binary measure comparing current e-cigarette users with non-current users. Non-current users comprised respondents who had used e-cigarettes in the past and those who had never used them. In the final analytic sample, 542 respondents were classified as current e-cigarette users and 12,755 as non-current users.

### Covariates

Several covariates were included to address potential confounding. These variables were selected a priori based on their established associations with tobacco use, nicotine product use, socioeconomic circumstances, and health in older populations [23–25]. Age was grouped into five-year categories: 60–64, 65–69, 70–74, 75–79, and 80 years or older. Sex was coded as male or female. Area deprivation was measured using the Scottish Index of Multiple Deprivation (SIMD) 2020 quintiles, ranging from most deprived (quintile 1) to least deprived (quintile 5). Marital status was classified as married or in a civil partnership, living as married, single, separated, divorced or dissolved civil partnership, and widowed or surviving civil partner.

Cigarette smoking status was categorised into current cigarette smoker, ex-smoker, and never smoked. For the regression analysis, never smoked was specified as the reference category to allow clearer interpretation of the relationship between smoking history and current e-cigarette use. Alcohol consumption was classified into four categories: never drank alcohol, ex-drinker, drinks within the UK Chief Medical Officers' low-risk drinking guidelines (up to 14 units per week), and drinks beyond these guidelines [26]. Self-rated general health was grouped into three levels: very good or good (reference category), fair, and bad or very bad. These covariates were harmonised across the pooled survey years to maximise comparability. There was no missing covariate information in the final analytic sample.

### Statistical analysis

All analyses were conducted using Stata version 18 (StataCorp, College Station, TX) and accounted for the complex survey design of the SHeS using Stata’s svy prefix commands. Survey weights, strata, and primary sampling units were incorporated to produce nationally representative estimates and correct standard errors for the clustered, stratified sampling design.

Descriptive statistics were used to characterise the study population by current e-cigarette use status. Unweighted counts and percentages were reported for the distribution of respondents across covariate categories, and the weighted prevalence of current e-cigarette use was estimated with 95% confidence intervals. Design-adjusted Rao–Scott F statistics were used to test for differences in the distribution of covariates between current and non-current e-cigarette users. Year-specific weighted prevalence estimates were calculated to examine trends in e-cigarette use over the study period. A formal linear trend test was conducted by including survey year as a continuous predictor in both unadjusted and adjusted survey-weighted logistic regression models; a further analysis excluded the 2021 wave, which was affected by pandemic-related disruption.

Survey-weighted logistic regression was used to estimate the association between each covariate and current e-cigarette use. Crude odds ratios were estimated in unadjusted models for each covariate separately. An adjusted model included all covariates simultaneously to identify independent correlates of current use. Odds ratios were reported with 95% confidence intervals. A subsidiary analysis restricted to current cigarette smokers examined the association between smoking intensity (light: fewer than 10 cigarettes per day; moderate: 10 to fewer than 20 per day; heavy: 20 or more per day) and current e-cigarette use, adjusting for all other covariates. All models additionally controlled for survey year to account for secular trends. Statistical significance was assessed at the conventional α = 0.05 level.

We also conducted several pre-specified sensitivity analyses. First, a multinomial logistic regression was fitted with a three-category outcome (never, former, and current e-cigarette use) to assess whether combining former and never users in the primary binary outcome obscured meaningful heterogeneity. Second, age was modelled as a continuous variable to verify that the categorical age gradient was not an artefact of categorisation., model diagnostics were assessed using variance inflation factors (VIF) from an equivalent ordinary-least-squares specification fitted to the same analytic sample, and model fit was examined using the Hosmer–Lemeshow test and decile-of-risk calibration based on an equivalent non-survey logistic model.

## Results

### Sample characteristics and prevalence of e-cigarette use

The final analytic sample comprised 13,297 older adults aged 60 years and over (mean age 71.4 years, range 60–102). The weighted prevalence of current e-cigarette use was 4.5% (95% CI: 4.1–4.9), corresponding to 542 current users and 12,755 non-current users. The majority of the sample was aged 60–74 (68.0%), female (54.3%), married or in a civil partnership (59.0%), and reported very good or good self-rated health (61.2%). Just over half had never smoked (51.4%), while 38.1% were ex-smokers and 10.4% were current cigarette smokers (Table 1).

**Table 1:** Unweighted sample characteristics and survey-weighted prevalence of current e-cigarette use among adults aged 60 years and over, Scottish Health Survey 2017, 2018, 2019, and 2021–2024 (n = 13,297)

Variable	Non-current user (n = 12,755)	Current user (n = 542)	Total (n = 13,297)	F, p-value
Weighted prevalence	95.5% (95% CI: 95.1 to 95.9)	4.5% (95% CI: 4.1 to 4.9)	100.0%	
Age group				F(3.97, 11,165.06) = 39.36, p < 0.001
60–64	2,817 (22.1%)	221 (40.8%)	3,038 (22.9%)	
65–69	2,942 (23.1%)	160 (29.5%)	3,102 (23.3%)	
70–74	2,810 (22.0%)	103 (19.0%)	2,913 (21.9%)	
75–79	2,070 (16.2%)	39 (7.2%)	2,109 (15.9%)	
80 +	2,116 (16.6%)	19 (3.5%)	2,135 (16.1%)	
Sex				F(1, 2809) = 0.62, p = 0.431
Male	5,819 (45.6%)	255 (47.1%)	6,074 (45.7%)	
Female	6,936 (54.4%)	287 (53.0%)	7,223 (54.3%)	
SIMD 2020 quintile				F(3.98, 11,190.14) = 24.71, p < 0.001
Most deprived	1,665 (13.1%)	137 (25.3%)	1,802 (13.6%)	
2nd	2,252 (17.7%)	133 (24.5%)	2,385 (17.9%)	
3rd	2,980 (23.4%)	137 (25.3%)	3,117 (23.4%)	
4th	3,208 (25.2%)	84 (15.5%)	3,292 (24.8%)	
Least deprived	2,650 (20.8%)	51 (9.4%)	2,701 (20.3%)	
Marital status				F(4.94, 13,868.42) = 12.19, p < 0.001
Married/civil partnership	7,571 (59.4%)	268 (49.5%)	7,839 (59.0%)	
Living as married	395 (3.1%)	26 (4.8%)	421 (3.2%)	
Single	816 (6.4%)	35 (6.5%)	851 (6.4%)	
Married/civil partnership – separated	229 (1.8%)	20 (3.7%)	249 (1.9%)	
Divorced/dissolved civil partnership	1,274 (10.0%)	106 (19.6%)	1,380 (10.4%)	
Widowed/surviving civil partner	2,470 (19.4%)	87 (16.1%)	2,557 (19.2%)	
Cigarette smoking status				F(1.99, 5601.63) = 217.94, p < 0.001
Current cigarette smoker	1,228 (9.6%)	159 (29.3%)	1,387 (10.4%)	
Ex-smoker	4,715 (37.0%)	356 (65.7%)	5,071 (38.1%)	
Never smoked	6,812 (53.4%)	27 (5.0%)	6,839 (51.4%)	
Alcohol status				F(2.98, 8357.86) = 3.60, p = 0.013
Never drank alcohol	1,071 (8.4%)	31 (5.7%)	1,102 (8.3%)	
Ex-drinker	1,758 (13.8%)	79 (14.6%)	1,837 (13.8%)	
Drinks within guidelines	5,762 (45.2%)	221 (40.8%)	5,983 (45.0%)	
Drinks beyond guidelines	4,164 (32.7%)	211 (38.9%)	4,375 (32.9%)	
Self-rated general health				F(2.00, 5606.89) = 38.94, p < 0.001
Very good/good	7,904 (62.0%)	238 (43.9%)	8,142 (61.2%)	
Fair	3,337 (26.2%)	175 (32.3%)	3,512 (26.4%)	
Bad/very bad	1,514 (11.9%)	129 (23.8%)	1,643 (12.4%)	

Weighted prevalence estimates of current e-cigarette use were derived using the Scottish Health Survey complex survey design, incorporating primary sampling units, strata, and sampling weights. Counts and percentages within each characteristic are unweighted and represent the distribution of respondents by current e-cigarette use status. Design-adjusted F statistics with Rao–Scott correction were used to test group differences. Totals represent complete case counts for each variable

Current e-cigarette users differed markedly from non-current users across most characteristics. Users were substantially younger: 40.8% were aged 60–64 compared with 22.1% of non-users, while only 3.5% of users were aged 80 or over compared with 16.6% of non-users (F[3.97, 11,165.06] = 39.36, *p* < 0.001). E-cigarette users were considerably more likely to reside in the most deprived areas (25.3% versus 13.1%) and less likely to reside in the least deprived areas (9.4% versus 20.8%; F[3.98, 11,190.14] = 24.71, *p* < 0.001). There was no significant difference by sex (F[1, 2809] = 0.62, *p* = 0.431). Alcohol consumption differed significantly between groups (F[2.98, 8357.86] = 3.60, *p* = 0.013). E-cigarette users were more likely to drink beyond government guidelines (38.9% versus 32.7%) and less likely to have never drunk alcohol (5.7% versus 8.4%).

The most striking distributional difference was in cigarette smoking status. Among current e-cigarette users, 29.3% were current cigarette smokers and 65.7% were ex-smokers, whereas only 5.0% had never smoked. By contrast, 53.4% of non-current users had never smoked (F[1.99, 5601.63] = 217.94, *p* < 0.001). E-cigarette users also reported poorer self-rated health: 23.8% reported bad or very bad health compared with 11.9% of non-users (F[2.00, 5606.89] = 38.94, *p* < 0.001).

### Trends over time

**Fig. 1:**
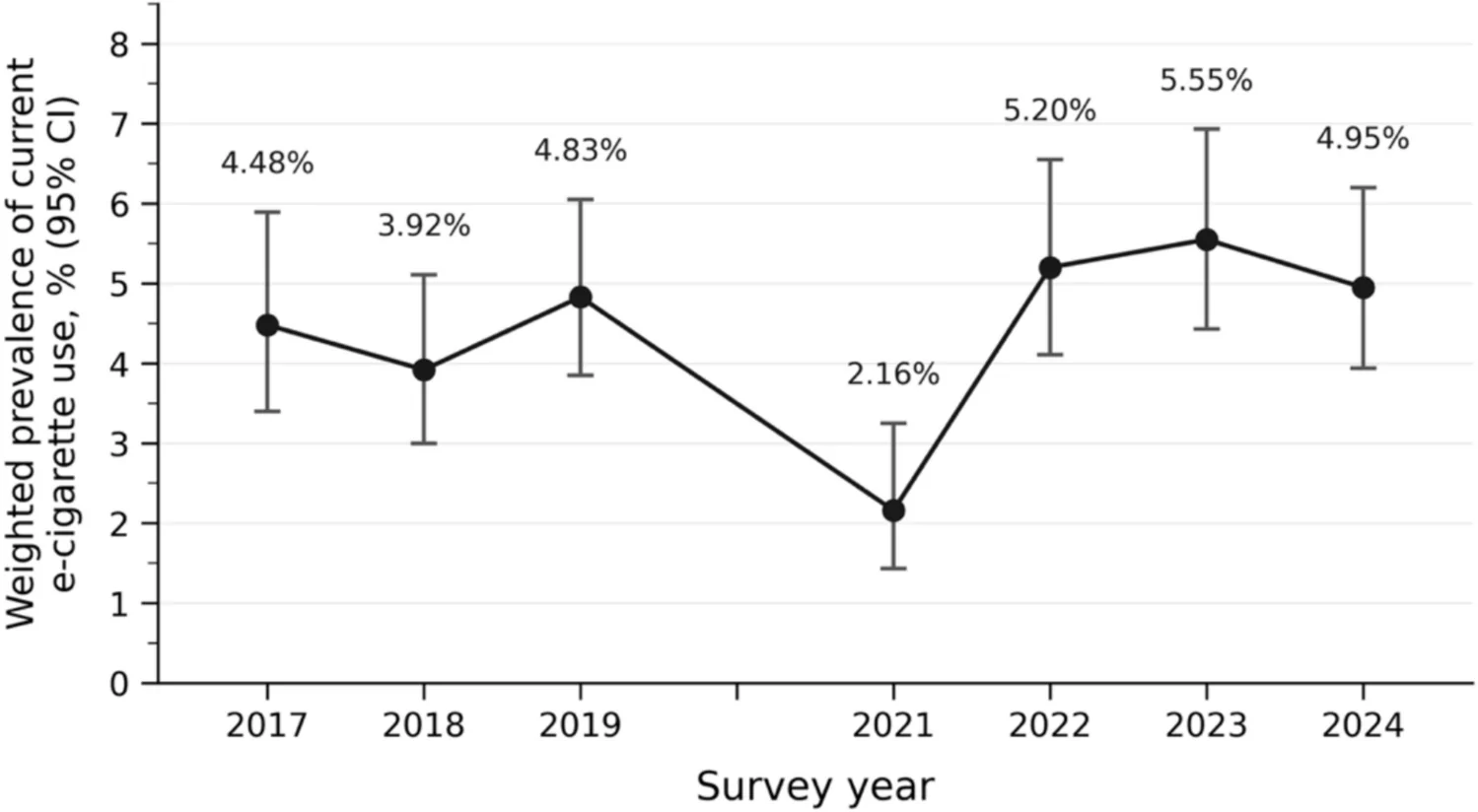
Year-specific survey-weighted prevalence of current e-cigarette use among adults aged 60 years and over, Scottish Health Survey 2017–2024. Weighted prevalence estimates were derived using the Scottish Health Survey complex survey design, incorporating sampling weights, strata, and primary sampling units. Estimates are restricted to adults aged 60 years and over

Year-specific weighted prevalence estimates revealed no consistent secular trend in current e-cigarette use over the study period (Fig. 1). Prevalence was 4.48% (95% CI: 3.40–5.89) in 2017, 3.92% (95% CI: 3.00–5.11) in 2018, 4.83% (95% CI: 3.85–6.05) in 2019, 2.16% (95% CI: 1.43–3.25) in 2021, 5.20% (95% CI: 4.11–6.55) in 2022, 5.55% (95% CI: 4.43–6.93) in 2023, and 4.95% (95% CI: 3.94–6.20) in 2024. The notably low estimate in 2021 likely reflects disruption associated with the COVID-19 pandemic. Excluding 2021, prevalence ranged from 3.92% to 5.55%, suggesting a broadly stable pattern with modest year-to-year fluctuation and a possible gentle upward trajectory in the post-pandemic period.

Formal trend testing provided mixed evidence of secular change. In an unadjusted survey-weighted model with survey year as a continuous predictor, there was no statistically significant linear trend in current e-cigarette use across survey years (OR per calendar year 1.03, 95% CI: 0.99–1.08, *p* = 0.136). After adjustment for sociodemographic, smoking, alcohol, and self-rated health covariates, survey year was associated with modestly higher odds of current e-cigarette use (adjusted OR per calendar year 1.06, 95% CI: 1.01–1.10, *p* = 0.019). However, this association was attenuated when the low-prevalence 2021 wave was excluded (OR per calendar year 1.03, 95% CI: 0.99–1.08, *p* = 0.095), suggesting that the apparent adjusted increase was substantially driven by the pandemic-affected 2021 estimate (See Supplementary **Table S3**).

### Crude and adjusted associations

Table 2 presents the results of the crude and adjusted logistic regression analyses. In the adjusted model, cigarette smoking status was the dominant correlate of current e-cigarette use. Compared with never smokers, current cigarette smokers had 18.78 times the odds of current e-cigarette use (95% CI: 11.79–29.92, *p* < 0.001) and ex-smokers had 20.17 times the odds (95% CI: 13.10–31.06, *p* < 0.001). The magnitude of these associations far exceeded that of any other covariate and underscores the centrality of smoking history in explaining e-cigarette use in this population.

**Table 2:** Crude and adjusted associations with current e-cigarette use among older adults aged 60 years and over

Variable	Crude OR (95% CI), p-value	Adjusted OR (95% CI), p-value
Age group		
60–64	Reference	Reference
65–69	0.65 (0.51–0.83), 0.001	0.61 (0.47–0.79), < 0.001
70–74	0.47 (0.36–0.61), < 0.001	0.44 (0.33–0.58), < 0.001
75–79	0.22 (0.15–0.32), < 0.001	0.19 (0.13–0.29), < 0.001
80 +	0.11 (0.07–0.18), < 0.001	0.11 (0.06–0.19), < 0.001
Sex		
Male	Reference	Reference
Female	0.93 (0.77–1.12), 0.431	0.98 (0.80–1.20), 0.857
SIMD 2020 quintile		
Most deprived	Reference	Reference
2nd	0.78 (0.59–1.02), 0.074	0.94 (0.70–1.25), 0.655
3rd	0.58 (0.44–0.77), < 0.001	0.79 (0.58–1.08), 0.136
4th	0.33 (0.24–0.46), < 0.001	0.49 (0.34–0.70), < 0.001
Least deprived	0.23 (0.16–0.33), < 0.001	0.41 (0.27–0.61), < 0.001
Marital status		
Married/civil partnership	Reference	Reference
Living as married	1.67 (1.01–2.74), 0.044	1.08 (0.62–1.87), 0.790
Single	1.28 (0.86–1.91), 0.228	0.98 (0.65–1.49), 0.928
Married/civil partnership – separated	2.24 (1.35–3.73), 0.002	1.34 (0.78–2.30), 0.289
Divorced/dissolved civil partnership	2.42 (1.86–3.15), < 0.001	1.65 (1.22–2.23), 0.001
Widowed/surviving civil partner	0.93 (0.70–1.23), 0.602	1.27 (0.92–1.73), 0.142
Cigarette smoking status		
Never smoked	Reference	Reference
Current cigarette smoker	31.36 (20.08–48.96), < 0.001	18.78 (11.79–29.92), < 0.001
Ex-smoker	19.64 (12.82–30.11), < 0.001	20.17 (13.10–31.06), < 0.001
Alcohol status		
Never drank alcohol	Reference	Reference
Ex-drinker	1.64 (1.02–2.64), 0.040	0.85 (0.51–1.40), 0.518
Drinks within guidelines	1.49 (0.95–2.34), 0.082	1.21 (0.75–1.94), 0.433
Drinks beyond guidelines	1.91 (1.22–2.99), 0.005	1.16 (0.72–1.88), 0.541
Self-rated general health		
Very good/good	Reference	Reference
Fair	1.63 (1.29–2.05), < 0.001	1.32 (1.03–1.70), 0.028
Bad/very bad	2.85 (2.24–3.61), < 0.001	1.73 (1.31–2.27), < 0.001

Crude and adjusted odds ratios (ORs) with 95% confidence intervals (CIs) were estimated using survey-weighted logistic regression models accounting for primary sampling units, strata, and sampling weights. The adjusted model additionally controlled for survey year, although survey year estimates are not shown in the table. Never smoked was specified as the reference category for cigarette smoking status

A clear and monotonic age gradient was observed. Taking the 60–64 age group as the reference, the adjusted odds of current e-cigarette use were 39% lower in the 65–69 group (OR 0.61, 95% CI: 0.47–0.79), 56% lower in the 70–74 group (OR 0.44, 95% CI: 0.33–0.58), 81% lower in the 75–79 group (OR 0.19, 95% CI: 0.13–0.29), and 89% lower in the 80 and over group (OR 0.11, 95% CI: 0.06–0.19). This gradient remained robust after adjustment for all other covariates, indicating that it is not attributable to confounding by smoking history, deprivation, or health status.

Area deprivation showed a significant gradient. Compared with the most deprived quintile, the adjusted odds of e-cigarette use were approximately 50% lower in the fourth quintile (OR 0.49, 95% CI: 0.34–0.70) and 59% lower in the least deprived quintile (OR 0.41, 95% CI: 0.27–0.61). The second and third quintiles did not differ significantly from the most deprived. This pattern indicates that the deprivation gradient is driven primarily by lower e-cigarette use in the most affluent areas rather than a stepwise gradient across all quintiles.

Poorer self-rated health was associated with higher odds of e-cigarette use. Compared with those reporting very good or good health, respondents reporting fair health had 32% higher adjusted odds (OR 1.32, 95% CI: 1.03–1.70, *p* = 0.028), and those reporting bad or very bad health had 73% higher odds (OR 1.73, 95% CI: 1.31–2.27, *p* < 0.001). This dose–response relationship persisted after adjustment for smoking status and deprivation, suggesting an independent contribution of subjective health to e-cigarette use.

Among marital status categories, only being divorced or having a dissolved civil partnership was significantly associated with current e-cigarette use (adjusted OR 1.65, 95% CI: 1.22–2.23, *p* = 0.001) compared with being married or in a civil partnership. No other marital status category reached statistical significance in the adjusted model. Sex was not associated with current e-cigarette use in either crude (OR 0.93, 95% CI: 0.77–1.12) or adjusted (OR 0.98, 95% CI: 0.80–1.20) analyses. Alcohol consumption was not independently associated with e-cigarette use after adjustment.

### Smoking intensity and e-cigarette use

**Fig. 2:**
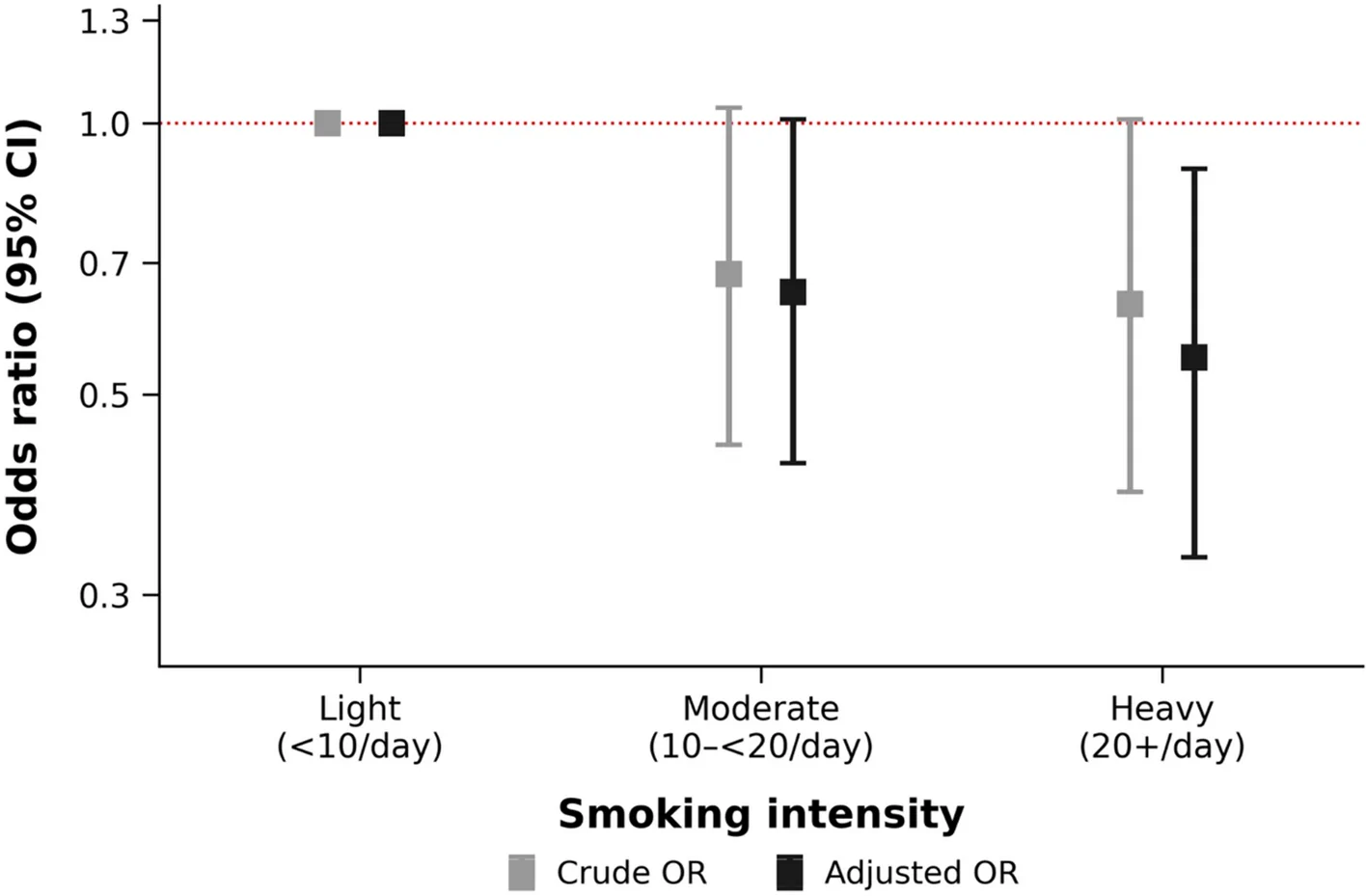
Smoking intensity and current e-cigarette use among current cigarette smokers aged 60 years and over. Crude and adjusted odds ratios (ORs) with 95% confidence intervals (CIs) were estimated using survey-weighted logistic regression restricted to current cigarette smokers aged 60 years and over. The adjusted model included age group, sex, SIMD quintile, marital status, alcohol status, self-rated general health, and survey year. The unweighted number of respondents in each smoking intensity category was as follows: light smokers (< 10 cigarettes/day), *n* = 350; moderate smokers (10 to < 20 cigarettes/day), *n* = 583; heavy smokers (20 + cigarettes/day), *n* = 404

In a subsidiary analysis restricted to current cigarette smokers (Fig. 2), heavier smoking intensity was not associated with higher odds of concurrent e-cigarette use; if anything, an inverse association was observed, with adjusted odds of e-cigarette use lower among heavy smokers (≥ 20 cigarettes/day) than among light smokers (< 10 cigarettes/day). This pattern may reflect deeper nicotine dependence or lower motivation to modify smoking behaviour among the heaviest smokers, although alternative explanations cannot be excluded in a cross-sectional design.

### Sensitivity analyses and model diagnostics

The principal findings were robust across pre-specified sensitivity analyses. In a multinomial model distinguishing never, former, and current e-cigarette users (Supplementary Table S1), the direction and magnitude of associations for current users versus never users were materially unchanged: current cigarette smokers (RRR 33.63, 95% CI: 20.99–53.87) and ex-smokers (RRR 21.39, 95% CI: 13.88–32.99) remained substantially more likely to be current e-cigarette users, and the inverse age gradient persisted across older age groups (RRR for 80 + vs 60–64: 0.09, 95% CI: 0.05–0.16). Former e-cigarette users showed broadly similar patterns to current users in their association with smoking history and age, although the magnitude of association with current cigarette smoking was even stronger for former e-cigarette users (RRR 220.46, 95% CI: 117.57–413.38), reflecting the very small number of never-smoker former vapers in the data. When age was modelled continuously rather than in five-year categories (Supplementary Table S2), each additional year of age was associated with approximately 9.7% lower odds of current e-cigarette use (adjusted OR per year 0.90, 95% CI: 0.89–0.92, *p* < 0.001), confirming the robustness of the categorical age gradient findings.

Multicollinearity diagnostics from an equivalent ordinary-least-squares specification fitted to the same analytic sample indicated no evidence of problematic collinearity, with a maximum variance inflation factor of 3.63 and a mean VIF of 1.79 (Supplementary Table S4). Model fit assessed using the Hosmer–Lemeshow test from an equivalent non-survey logistic regression showed no evidence of poor fit (χ^2^[8] = 3.21, *p* = 0.921), and observed and predicted probabilities were closely aligned across deciles of predicted risk (Supplementary Table S5). Taken together, these sensitivity analyses support the robustness of the primary findings and indicate that the conclusions are not driven by the choice of outcome categorisation, the parameterisation of age, or by collinearity or poor fit in the adjusted model.

## Discussion

This study provides one of the first dedicated analyses of the prevalence and correlates of e-cigarette use among older adults in Scotland. Using pooled data from seven waves of the Scottish Health Survey encompassing 13,297 adults aged 60 years and over, we found a weighted prevalence of current e-cigarette use of 4.5%. Year-specific weighted prevalence remained broadly stable between 2017 and 2024, with a transient decline in 2021 attributable to pandemic-related disruption; formal trend testing provided mixed evidence of secular change. The dominant correlate of e-cigarette use was cigarette smoking history: current and ex-smokers had approximately 19–20 times the odds of current use compared with never smokers. E-cigarette use declined sharply with advancing age, was concentrated in more deprived areas, and was associated with poorer self-rated health. Sex and alcohol consumption were not independently associated with use.

The 4.5% prevalence observed in our study is consistent with, though somewhat higher than, estimates reported in other high-income countries. In England, data from a large cross-sectional household survey spanning 2014 to 2024 found that vaping among adults aged 65 and over remained below 4% [27], while in the United States, nationally representative estimates indicate that current e-cigarette use is relatively low among older adults, at 3.3% among those aged 50–64 years and 0.9% among those aged 65 years and older in 2023 [28]. The slightly higher prevalence in Scotland may reflect the relatively supportive policy environment towards e-cigarettes in the United Kingdom.

The strong concentration of e-cigarette use among current and former smokers is a central finding of this study and aligns with the broader literature. In our sample, 95% of current e-cigarette users had a history of cigarette smoking, and only 5% were never smokers. This pattern is consistent with use among individuals with prior smoking history and may suggest that, in this age group, e-cigarette use occurs predominantly after exposure to combustible tobacco rather than as a route of primary nicotine initiation. The cross-sectional design, however, cannot establish motivation, timing, or directionality, and the data do not include information on quit intentions, duration of e-cigarette use, dependence severity, or the timing of smoking cessation relative to vaping initiation. The strong association with smoking history is therefore compatible with several scenarios, including transition away from combustible cigarettes, persistent dual use, or maintenance of nicotine dependence after smoking cessation. The subsidiary finding that heavier smokers were less likely to use e-cigarettes concurrently is consistent with the possibility that vaping is more readily adopted among lighter smokers or those further along a cessation trajectory, but alternative explanations – including deeper nicotine dependence among heavier smokers – cannot be excluded [29].

The steep age gradient observed within the older adult population deserves particular attention from a geriatric perspective. Although all participants were aged 60 or over, the adjusted odds of e-cigarette use among those aged 80 and over were 89% lower than among those aged 60–64, and the gradient was confirmed in continuous-age sensitivity analyses (approximately 9.7% lower odds per additional year of age). This gradient is unlikely to be fully explained by differences in smoking history, as it persisted after adjustment for smoking status. Several mechanisms specific to the oldest-old population are plausible. First, cohort effects: those currently aged 80 and over reached older age before e-cigarettes were widely available, established their smoking or cessation patterns decades earlier, and are less likely to have encountered vaping in their social or healthcare environments. Second, functional barriers: e-cigarette devices require fine motor coordination for refilling, replacing coils, and operating buttons or touch-activated interfaces; reduced manual dexterity, declining vision, and cognitive impairment may make these tasks impractical for many adults in the oldest-old age band, even where motivation exists. Third, care-dependency environments: a substantial proportion of the oldest-old live in supported accommodation, sheltered housing, or care settings where access to vaping products, charging facilities, and discretionary expenditure may be constrained, and where staff guidance on e-cigarettes is often absent or cautious. Fourth, differential survivorship: heavy smokers may not survive to the oldest age bands, and surviving never smokers and quitters may have less reason to adopt nicotine-containing products. Together, these factors suggest that very low uptake among the oldest-old reflects a combination of cohort, functional, environmental, and selection effects rather than a uniform absence of interest. Research from England and the United States further indicates that older adults often hold less accurate perceptions of the relative harms of e-cigarettes compared with cigarettes, and that these misperceptions are associated with lower e-cigarette use or reduced switching from smoking [30, 31].

The socioeconomic gradient in e-cigarette use observed in this study, with higher use in more deprived areas, contrasts with findings from some adolescent studies in which greater affluence has been linked to higher exposure to e-cigarette marketing and more frequent use, although evidence in younger populations remains mixed [32, 33]. Among older adults, the association between deprivation and e-cigarette use likely reflects the strong socioeconomic gradient in smoking itself: smoking prevalence is highest in the most deprived communities [29], and e-cigarette use in this age group is almost entirely derivative of smoking history. This interpretation is supported by the attenuation of the crude deprivation gradient after adjustment for smoking status, though the gradient remained statistically significant, suggesting that deprivation captures additional variance beyond smoking history alone.

The independent association between poorer self-rated health and current e-cigarette use, after adjustment for smoking status and deprivation, has several plausible explanations that the cross-sectional design cannot disentangle. First, reverse causation: experiencing the health consequences of smoking first hand – chronic respiratory symptoms, cardiovascular events, or a smoking-related diagnosis – may prompt some older adults to switch to or take up e-cigarette use, in which case worse self-rated health precedes vaping rather than the reverse [34]. Second, residual confounding by lifetime smoking exposure: a three-category smoking status variable (current, ex-, never) does not fully capture pack-years, duration of smoking, dependence severity, or cumulative tar and nicotine exposure, all of which independently influence both self-rated health and the likelihood of trying e-cigarettes; the observed health association may therefore in part reflect a “sicker smokers transitioning” phenotype rather than an independent effect of poor health on vaping uptake. Third, shared upstream determinants: poorer mental health, social disadvantage, multimorbidity, and reduced access to formal cessation support may simultaneously elevate the risk of poor self-rated health and increase the likelihood of seeking alternative nicotine products outside conventional pathways. Fourth, the direct adverse effects of nicotine and aerosol exposure on perceived health among current vapers cannot be excluded. These interpretations are not mutually exclusive, and the direction of causality cannot be determined in a cross-sectional design.

These findings carry several implications for policy and practice. First, the low prevalence of e-cigarette use among older adults, combined with the persistently high burden of smoking-related disease in this population, suggests that older adults represent a neglected demographic in tobacco harm reduction efforts. Public health agencies should consider whether existing cessation support and harm reduction messaging are reaching older adults effectively, or whether they are implicitly designed for and delivered to younger populations.

Second, the strong association between smoking history and e-cigarette use indicates that, in this population, current vaping is largely confined to people with prior exposure to combustible tobacco. This pattern is compatible with use as a partial substitute for cigarettes among some older smokers, but the data cannot determine the proportion of users who have transitioned completely from smoking, those who continue to use both products, and those whose vaping serves primarily to maintain nicotine dependence. Decisions about facilitating access to e-cigarettes for older adults should therefore weigh the potential reduction in combustion-related harm against the uncertainties of long-term vaping effects in this age group, the persistence of nicotine dependence, and the risk of sustained dual use. Where switching is being considered, clinicians should help patients distinguish clearly between complete cessation of combustible tobacco and concurrent use of both products.

Third, the deprivation gradient in e-cigarette use highlights the risk that harm reduction benefits may accrue disproportionately to more deprived communities. While this is desirable from an equity perspective, it also means that if e-cigarettes carry residual health risks, those risks would also be concentrated in already disadvantaged populations. Balanced, evidence-based public health communication is needed to ensure that older adults in all socioeconomic strata can make informed decisions about nicotine use.

Fourth, the misperception of e-cigarettes as equally or more harmful than combustible cigarettes, documented extensively in older populations, represents a modifiable barrier to switching. Health communications targeted at older adults could address these misperceptions while maintaining appropriate caveats about remaining scientific uncertainties [9]. Healthcare providers, particularly those working in primary care and geriatric medicine, are well positioned to deliver accurate, personalised advice about the relative risks of continued smoking versus switching to e-cigarettes.

This study has several strengths. It uses seven waves of a nationally representative household survey with a large pooled sample of over 13,000 older adults, providing robust estimates and adequate statistical power to detect moderate associations. The survey-weighted analytical approach accounts for the complex sampling design, producing estimates that are generalisable to the Scottish population. The inclusion of multiple survey years allows examination of temporal trends, and the pooled design maximises sample size in a population where the behaviour of interest is relatively uncommon.

From a geriatric medicine perspective, these findings suggest that nicotine product use should be assessed as part of routine smoking history taking in older adults, particularly among patients with multimorbidity, respiratory or cardiovascular disease, frailty, or poor self-rated health. Clinicians should avoid assuming that current e-cigarette use necessarily represents successful cessation or reduced overall risk, because persistent dual use and continued nicotine dependence may be common. Counselling should distinguish clearly between (i) complete smoking cessation with no nicotine product use, (ii) complete switching from combustible cigarettes to e-cigarettes, and (iii) concurrent use of both products, and should be tailored to the patient’s clinical picture and treatment goals. In older adults, discussions about vaping should also take account of device handling in the context of arthritis, tremor, or reduced visual acuity; cognitive capacity to follow device-maintenance instructions; medication burden and potential interactions; respiratory symptoms and existing airway disease; cardiovascular risk including arrhythmia history; and the patient’s care and support environment. Where switching is being considered, integration with established geriatric assessment pathways, frailty screening, and falls-risk evaluation may help individualise advice and identify situations in which the practical, cognitive, or environmental demands of device use may outweigh potential benefit. The very low prevalence observed in the oldest-old group should not be interpreted as evidence that e-cigarettes are unimportant in this population, but rather as a signal that any clinical discussion in this group must be especially attentive to functional capacity, decision-making, and goals of care.

A balanced consideration of harms is particularly important when interpreting these findings in an older population. Although the toxicant profile of e-cigarette aerosol is substantially less complex than that of combustible tobacco smoke, e-cigarettes are not risk free [35]. Evidence to date raises concerns about acute and longer-term cardiovascular effects, including changes in blood pressure, heart rate variability, and endothelial function; respiratory effects including cough, airway irritation, and potential exacerbation of pre-existing airway disease; persistence of nicotine dependence with its own cardiovascular and metabolic implications; and uncertainty about long-term consequences of chronic aerosol exposure that has not yet been observed over a typical older adult lifetime [36–38]. These concerns may be amplified in older adults by underlying multimorbidity, reduced physiological reserve, polypharmacy, and frailty-related vulnerability. The balance of benefit and harm for an individual older smoker therefore depends on their current smoking status, willingness and ability to quit by other means, comorbid disease, and the realistic alternative pathways available to them. Population-level messaging should reflect this nuance and avoid both unduly alarmist and unduly reassuring framings.

Several limitations should be acknowledged. First, the cross-sectional design precludes causal inference; observed associations may be subject to reverse causation or residual confounding, and the design cannot determine whether e-cigarette use among older adults represents cessation, substitution, experimentation, or persistent dual use. In particular, the relationship between self-rated health and e-cigarette use may be bidirectional. Second, e-cigarette use was ascertained by self-report, which may be subject to recall bias or social desirability bias, although the confidential nature of the survey should mitigate these concerns. Third, smoking exposure was captured using a three-category variable (current, ex-, never smoker), which does not capture pack-years, duration of smoking, recency of cessation, cumulative nicotine exposure, dependence severity, or prior quit attempts. The Scottish Health Survey does not consistently collect these dimensions in the age range of interest, and we were therefore unable to adjust for the full extent of cumulative smoking exposure. This is particularly relevant when interpreting the association between poorer self-rated health and e-cigarette use, which may in part reflect heavier lifetime smoking exposure among users. Fourth, the SHeS does not collect information on quit intentions, duration of e-cigarette use, timing of vaping initiation relative to smoking cessation, motivations for vaping, dual-use duration, nicotine dependence severity, or use of pharmacological cessation aids, limiting our ability to characterise the function of e-cigarettes in this population beyond the descriptive level.

Fifth, the primary outcome combined former and never e-cigarette users into a single non-current category. While this provides a clinically meaningful contrast for current vaping, former and never users may differ in smoking history, nicotine dependence, and health behaviours; a multinomial sensitivity analysis (Supplementary Table S1) partially addressed this concern and produced consistent findings, but residual heterogeneity within the non-current category cannot be excluded. Sixth, the exclusion of the 2020 wave due to pandemic disruption introduces a gap in the time series, and the unusually low 2021 estimate likely reflects continued pandemic-related disruption rather than a true secular shift. Seventh, small cell sizes for some subgroups, particularly never-smoker former vapers and the oldest age categories, may limit the precision of estimates and restrict the detection of associations in subsidiary and multinomial analyses. Eighth, the Scottish Health Survey does not include validated geriatric measures of frailty, comprehensive geriatric assessment scores, cognitive function, or detailed multimorbidity counts, so we were unable to directly examine the relationship between vaping and these geriatric domains; this is an important direction for future work.

## Conclusion

Current e-cigarette use among Scottish adults aged 60 years and over was low but non-trivial, with a survey-weighted prevalence of 4.5%, and was strongly patterned by cigarette smoking history. The concentration of use among current and former smokers is consistent with vaping occurring predominantly among older adults with prior exposure to combustible tobacco, but the cross-sectional design cannot determine whether use reflects cessation, substitution, experimentation, or persistent dual use, and the data available do not include the duration, motivation, or dependence dimensions that would be required to make such inferences. The sharp decline in use with advancing age, the concentration of use in more deprived areas, and the independent association with poorer self-rated health highlight important inequalities and clinically meaningful patterns in this population. For geriatric practice, the findings argue for routine assessment of nicotine product use as part of smoking history taking in older adults, balanced risk communication that neither overstates nor dismisses potential benefit, and counselling that distinguishes complete cessation from switching and from dual use, with explicit attention to multimorbidity, frailty, cognition, dexterity, and care environment. Future research should employ longitudinal designs to examine transitions between smoking and e-cigarette use in older populations, link vaping status to validated measures of frailty, multimorbidity, and functional status, and evaluate the long-term health consequences of vaping and dual use in older age.

## Supplementary Information

Below is the link to the electronic supplementary material.

Supplementary file1 (DOCX 28 KB)

## Data Availability

The Scottish Health Survey data are available from the UK Data Service (https://ukdataservice.ac.uk).
