# Supplementary material for: Prevalence and correlates of e-cigarette use among older adults aged 60 years and over
Source: Eur Geriatr Med. 2026 Jun 29;17(4):2109–21. doi: 10.1007/s41999-026-01539-2 (PMC13562102; doi:10.1007/s41999-026-01539-2)
Supplement: Supplementary file 1 [file 41999_2026_1539_MOESM1_ESM.docx]

**Supplementary Material**

**Prevalence and Correlates of E-Cigarette Use Among Older Adults Aged 60 Years and Over**

**Supplementary Table S1. Survey-weighted multinomial logistic regression of e-cigarette use status (never, former, current) among adults aged 60 years and over, Scottish Health Survey 2017–2024 (n = 13,297).**

*Outcome categories: never e-cigarette user (n = 12,100; reference), former e-cigarette user (n = 655), and current e-cigarette user (n = 542). Estimates are relative risk ratios (RRR) from a single multinomial logistic regression model fitted using Stata's svy: mlogit with baseoutcome = never. The model adjusts for age group, sex, SIMD 2020 quintile, marital status, cigarette smoking status, alcohol status, self-rated general health, and survey year. *The RRR of 220.46 for current cigarette smokers in the former vs never e-cigarette user contrast reflects a very small number of never-smoker former vapers in the data; the wide confidence interval (117.57–413.38) should be interpreted with caution.*

| **Variable** | **Former vs never RRR** | **95% CI** | **p** | **Current vs never RRR** | **95% CI** | **p** |
| --- | --- | --- | --- | --- | --- | --- |
| **Age group** |  |  |  |  |  |  |
| 60–64 | Reference | — | — | Reference | — | — |
| 65–69 | 0.79 | 0.61–1.03 | 0.088 | 0.59 | 0.45–0.77 | <0.001 |
| 70–74 | 0.61 | 0.46–0.82 | 0.001 | 0.40 | 0.30–0.53 | <0.001 |
| 75–79 | 0.36 | 0.25–0.51 | <0.001 | 0.17 | 0.11–0.26 | <0.001 |
| 80+ | 0.12 | 0.07–0.21 | <0.001 | 0.09 | 0.05–0.16 | <0.001 |
| **Sex** |  |  |  |  |  |  |
| Male | Reference | — | — | Reference | — | — |
| Female | 0.89 | 0.73–1.08 | 0.244 | 0.96 | 0.78–1.17 | 0.676 |
| **SIMD quintile** |  |  |  |  |  |  |
| Most deprived | Reference | — | — | Reference | — | — |
| 2nd | 1.23 | 0.90–1.68 | 0.190 | 0.97 | 0.72–1.31 | 0.842 |
| 3rd | 0.96 | 0.69–1.32 | 0.784 | 0.79 | 0.58–1.07 | 0.132 |
| 4th | 0.94 | 0.67–1.32 | 0.727 | 0.49 | 0.34–0.70 | <0.001 |
| Least deprived | 0.67 | 0.45–1.00 | 0.053 | 0.40 | 0.26–0.59 | <0.001 |
| **Marital status** |  |  |  |  |  |  |
| Married/civil partnership | Reference | — | — | Reference | — | — |
| Living as married | 1.20 | 0.64–2.24 | 0.574 | 1.11 | 0.63–1.97 | 0.717 |
| Single | 0.89 | 0.59–1.34 | 0.570 | 0.95 | 0.62–1.46 | 0.824 |
| Separated | 1.20 | 0.68–2.14 | 0.527 | 1.39 | 0.80–2.40 | 0.238 |
| Divorced/dissolved | 1.38 | 1.03–1.85 | 0.032 | 1.77 | 1.31–2.39 | <0.001 |
| Widowed/surviving partner | 1.07 | 0.78–1.46 | 0.670 | 1.27 | 0.92–1.75 | 0.141 |
| **Cigarette smoking status** |  |  |  |  |  |  |
| Never smoked | Reference | — | — | Reference | — | — |
| Current cigarette smoker* | 220.46 | 117.57–413.38 | <0.001 | 33.63 | 20.99–53.87 | <0.001 |
| Ex-smoker | 18.62 | 9.93–34.92 | <0.001 | 21.39 | 13.88–32.99 | <0.001 |
| **Alcohol status** |  |  |  |  |  |  |
| Never drank alcohol | Reference | — | — | Reference | — | — |
| Ex-drinker | 0.97 | 0.56–1.67 | 0.910 | 0.84 | 0.51–1.40 | 0.511 |
| Within guidelines | 1.04 | 0.62–1.76 | 0.878 | 1.22 | 0.75–1.98 | 0.428 |
| Beyond guidelines | 0.94 | 0.56–1.60 | 0.825 | 1.15 | 0.70–1.89 | 0.568 |
| **Self-rated general health** |  |  |  |  |  |  |
| Very good/good | Reference | — | — | Reference | — | — |
| Fair | 1.22 | 0.94–1.58 | 0.133 | 1.36 | 1.05–1.75 | 0.018 |
| Bad/very bad | 1.66 | 1.23–2.24 | 0.001 | 1.90 | 1.44–2.50 | <0.001 |
| **Survey year** |  |  |  |  |  |  |
| 2017 | Reference | — | — | Reference | — | — |
| 2018 | 1.57 | 1.05–2.36 | 0.028 | 0.98 | 0.63–1.51 | 0.916 |
| 2019 | 1.54 | 1.02–2.32 | 0.038 | 1.22 | 0.81–1.82 | 0.336 |
| 2021 | 1.77 | 1.14–2.74 | 0.011 | 0.58 | 0.35–0.97 | 0.039 |
| 2022 | 1.45 | 0.97–2.18 | 0.071 | 1.35 | 0.89–2.03 | 0.155 |
| 2023 | 1.33 | 0.88–1.99 | 0.172 | 1.46 | 0.98–2.17 | 0.061 |
| 2024 | 1.28 | 0.81–2.00 | 0.289 | 1.40 | 0.93–2.12 | 0.111 |

**Supplementary Table S2. Sensitivity analysis modelling age as a continuous variable: survey-weighted logistic regression of current vs non-current e-cigarette use (n = 13,297).**

*Estimates are adjusted odds ratios (OR) from a single survey-weighted logistic regression model in which age was modelled continuously rather than in five-year categories. All other covariates are specified as in the primary adjusted model. Each one-year increase in age was associated with approximately 9.7% lower odds of current e-cigarette use (1 − 0.9027 = 0.0973).*

| **Variable** | **Adjusted OR** | **95% CI** | **p-value** |
| --- | --- | --- | --- |
| Age, per one-year increase | 0.90 | 0.89–0.92 | <0.001 |
| Female vs male | 0.98 | 0.80–1.19 | 0.813 |
| SIMD 2nd vs most deprived | 0.95 | 0.71–1.28 | 0.748 |
| SIMD 3rd vs most deprived | 0.80 | 0.59–1.09 | 0.158 |
| SIMD 4th vs most deprived | 0.49 | 0.34–0.71 | <0.001 |
| Least deprived vs most deprived | 0.41 | 0.27–0.61 | <0.001 |
| Living as married vs married/civil partnership | 1.03 | 0.59–1.79 | 0.911 |
| Single vs married/civil partnership | 0.97 | 0.64–1.48 | 0.903 |
| Separated vs married/civil partnership | 1.32 | 0.77–2.26 | 0.307 |
| Divorced/dissolved vs married/civil partnership | 1.66 | 1.23–2.24 | 0.001 |
| Widowed/surviving partner vs married/civil partnership | 1.31 | 0.95–1.79 | 0.095 |
| Current cigarette smoker vs never smoker | 18.92 | 11.89–30.12 | <0.001 |
| Ex-smoker vs never smoker | 20.35 | 13.20–31.38 | <0.001 |
| Ex-drinker vs never drank | 0.86 | 0.52–1.42 | 0.556 |
| Within guidelines vs never drank | 1.22 | 0.75–1.96 | 0.422 |
| Beyond guidelines vs never drank | 1.16 | 0.72–1.88 | 0.547 |
| Fair vs very good/good health | 1.32 | 1.03–1.70 | 0.029 |
| Bad/very bad vs very good/good health | 1.71 | 1.30–2.24 | <0.001 |
| 2018 vs 2017 | 0.90 | 0.58–1.37 | 0.615 |
| 2019 vs 2017 | 1.13 | 0.76–1.67 | 0.550 |
| 2021 vs 2017 | 0.54 | 0.32–0.90 | 0.018 |
| 2022 vs 2017 | 1.25 | 0.83–1.86 | 0.282 |
| 2023 vs 2017 | 1.40 | 0.95–2.06 | 0.092 |
| 2024 vs 2017 | 1.33 | 0.89–1.99 | 0.170 |

**Supplementary Table S3. Formal linear trend tests for current e-cigarette use across survey years.**

*Trend tests were conducted by including survey year as a continuous predictor in survey-weighted logistic regression models. The adjusted model controlled for age group, sex, SIMD quintile, marital status, cigarette smoking status, alcohol status, and self-rated general health. The third row presents the unadjusted trend test restricted to the subpopulation of survey years 2017–2019 and 2022–2024 (excluding the pandemic-affected 2021 wave), implemented using Stata's svy, subpop() option. *Adjusted estimate.*

| **Model** | **OR per year** | **95% CI** | **p-value** | **Interpretation** |
| --- | --- | --- | --- | --- |
| Unadjusted survey-weighted trend | 1.03 | 0.99–1.08 | 0.136 | No statistically significant unadjusted linear trend |
| Adjusted survey-weighted trend* | 1.06 | 1.01–1.10 | 0.019 | Modest adjusted increase over time |
| Unadjusted trend excluding 2021 | 1.03 | 0.99–1.08 | 0.095 | Attenuated; not statistically significant at α=0.05 |

**Supplementary Table S4. Multicollinearity diagnostics using variance inflation factors.**

*Variance inflation factors (VIF) were obtained from an equivalent ordinary-least-squares model fitted to the same analytic sample and predictor specification as the primary survey-weighted logistic regression model. For each multi-category predictor, the highest VIF among the dummy variables is reported. A maximum VIF of 3.63 and a mean VIF of 1.79 indicate no evidence of problematic multicollinearity.*

| **Predictor group** | **Highest VIF in group** |
| --- | --- |
| Age group | 1.71 |
| Sex | 1.07 |
| SIMD quintile | 2.27 |
| Marital status | 1.29 |
| Cigarette smoking status | 1.18 |
| Alcohol status | 3.63 |
| Self-rated general health | 1.18 |
| Survey year | 2.17 |
| **Mean VIF** | **1.79** |

**Supplementary Table S5. Model fit and calibration diagnostics.**

**Table S5a. Hosmer–Lemeshow goodness-of-fit test from equivalent non-survey logistic model.**

| **Diagnostic** | **Value** |
| --- | --- |
| Number of observations | 13,297 |
| Number of groups (deciles of risk) | 10 |
| Hosmer–Lemeshow χ² | 3.21 |
| Degrees of freedom | 8 |
| p-value | 0.921 |
| Interpretation | No evidence of poor model fit |

*The Hosmer–Lemeshow test was conducted on an equivalent non-survey logistic regression model fitted to the same analytic sample, since the test is not directly available within Stata's survey-weighted estimation framework. The non-significant test (p = 0.921) provides no evidence of poor model fit.*

**Table S5b. Observed and predicted current e-cigarette use by decile of predicted risk.**

| **Risk decile** | **Observed current e-cigarette use, %** | **Predicted probability, %** | **n** |
| --- | --- | --- | --- |
| 1 | 0.15 | 0.07 | 1,332 |
| 2 | 0.08 | 0.16 | 1,331 |
| 3 | 0.23 | 0.28 | 1,327 |
| 4 | 0.38 | 0.46 | 1,329 |
| 5 | 0.90 | 0.77 | 1,330 |
| 6 | 1.65 | 1.65 | 1,330 |
| 7 | 3.16 | 3.51 | 1,329 |
| 8 | 6.76 | 6.25 | 1,331 |
| 9 | 9.41 | 10.13 | 1,329 |
| 10 | 18.06 | 18.76 | 1,329 |
| **Total** | **4.08** | **4.20** | **13,297** |

*Observed proportions are derived from the analytic sample (n = 13,297). Predicted probabilities are derived from the primary survey-weighted adjusted logistic regression model. Observed and predicted probabilities are closely aligned across deciles of predicted risk, supporting acceptable model calibration.*
